# A rapid and simple method to quantify per- and polyfluoroalkyl substances (PFAS) in plasma and serum using 96-well plates

**DOI:** 10.1016/j.mex.2020.101111

**Published:** 2020-10-17

**Authors:** Bianca Ferreira Da Silva, Atiye Ahmadireskety, Juan J. Aristizabal-Henao, John A. Bowden

**Affiliations:** a College of Veterinary Medicine, Department of Physiological Sciences, University of Florida, Gainesville, FL, USA; b Department of Chemistry, University of Florida, Gainesville, FL, USA

**Keywords:** Biological samples, UHPLC-MS/MS, SRM 1950, PFAS, 96-well plate design

## Abstract

Per- and polyfluoroalkyl substances (PFAS) are synthetic organic compounds that over the past several years, have witnessed a dramatic increase in scientific attention. As PFAS are predominantly accumulated in plasma, monitoring individual burden levels in plasma are typically achieved via some combination of protein precipitation and/or solid phase extraction (SPE), either in online or offline modes. This work describes an updated PFAS extraction workflow, using 96-well plate technology and protein precipitation that is rapid, simple, inexpensive, and amenable for large cohort studies. In brief, plasma proteins were precipitated using methanol and the resulting centrifuged supernatant was directly analyzed using UHPLC-MS/MS. We monitored 51 PFAS, which were quantified via isotope dilution and the effectiveness of the method was demonstrated by using NIST blood-based Standard Reference Materials (SRMs). This method resulted in recoveries ranging between 70 and 89% for all analytes. The 96-well design exhibited low limits of detection and only required sample volumes of 100 µL, thus resulting in an amenable method for high-throughput plasma/serum PFAS screening.

• PFAS were directly quantified in plasma and serum samples;

• No SPE needed after protein precipitation;

• SRMs can be used to validate PFAS measurement in plasma/serum.

## Graphical abstract

**Figure:**
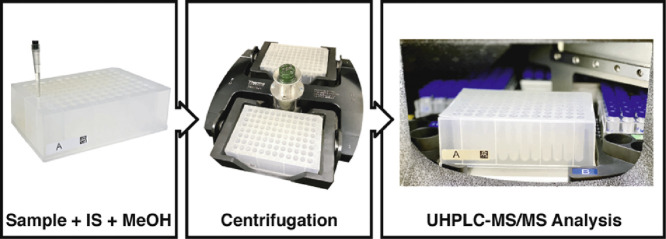

Specifications table

**Table:** 

Subject Area:	Chemistry
More specific subject area:	Analytical Chemistry
Method name:	Direct analysis of PFAS in biological samples using 96-well plates and protein precipitation
Name and reference of original method:	Flaherty, J. M.; Connolly, P. D.; Decker, E. R.; Kennedy, S. M.; Ellefson, M. E.; Reagen, W. K.; Szostek, B. [1]: Quantitative Determination of Perfluorooctanoic Acid in Serum and Plasma by Liquid Chromatography Tandem Mass Spectrometry. *J. Chromatogr. B Anal. Technol. Biomed. Life Sci.***2005**, *819* (2), 329–338. https://doi.org/10.1016/j.jchromb.2005.03.002
Resource availability:	Water (P/N: W6-4), methanol (P/N: A456-4), acetonitrile (P/N: A955-4), ammonium acetate (P/N: A114-50)– Optima Grade (Fisher Scientific) Mixture of 51 non-labeled (native) PFAS (Wellington Laboratories) made from PFAC-24PAR and individual standards (in methanol). For list (including abbreviations), see supplemental Table S1. Mixture 23 mass-labeled PFAS (Wellington Laboratories) made from MPFAC-24ES) and individual standards (in methanol). For list (including abbreviations), see supplemental Table S1. 96-well plates (7 mm RND, U base, 1.0 mL, PP, barcoded, P/N: 60,180-P201B) and Micromats (silicone, 7 mm H, round well shape, 96-well, pre-slit, P/N: 60,180-M113) were purchased from Thermo Scientific. National Institute of Standards and Technology (NIST) Standard Reference Material (SRM) 1950 – Metabolites in Frozen Human Plasma (NIST) and SRM 971 – Hormones in Frozen Human Serum (male and female vials), along with an in-house plasma pool collected from several adult American alligators (*Alligator mississippiensis*) from the Yawkey Wildlife Refuge in Charleston, SC (work done under the permit of the South Carolina Department of Natural Resources), were used to demonstrate the method. Equipment used with the method include: a Fisher microplate vortexer (120 V, ADV, Fisher Scientific), a Sorvall ST16R centrifuge (Thermo Scientific), and a Vanquish UHPLC (ultra-high pressure liquid chromatography) coupled to a TSQ Quantis triple quadrupole mass spectrometer (Thermo Scientific).

## Method details

### Background information and method applicability

Per- and polyfluoroalkyl substances (PFAS) are an anthropogenic chemical class of emerging concern [1], [2], [3]. Due to their unique structural properties, PFAS are highly persistent in the environment and bioaccumulate and biomagnify through the food chain. These compounds are present in a wide variety of consumer products, including but not limited to fast food packing, textiles, clothing, pesticides, firefighting foams and stain resistant materials [1,[3], [4], [5], [6]. Once an individual is exposed to these chemicals, these chemicals bind to blood proteins and accumulate in the blood and blood-rich tissues, such as the kidney and liver [4], [5], [6], [7], [8], [9], [10], [11], [12], [13]. For biological interrogation of PFAS exposure, analyses are typically carried out in blood matrices (e.g., plasma), blood rich tissues (e.g., liver); however, PFAS have been readily detected in other biological matrices, as well as several types of environmental matrices, including surface water, soil and sediments [14]. The predominant analytical approach to measure PFAS in plasma typically involves the use of ultra-high pressure liquid chromatography and tandem mass spectrometry (UHPLC-MS/MS); more specifically, triple quadrupole instrumentation utilizing selected reaction monitoring scanning modalities due to their high sensitivity and selectivity. To extract PFAS from blood-based liquid matrices, such as plasma or serum, most methods employ a protein crash (using an organic solvent, such as methanol or acetonitrile) followed by solid-phase extraction (SPE) [15], [16], [17], [18], [19]. However, many of these approaches do not use 96-well plate design and are high throughput limited (both by total cost and time) and thus, would limit their applicability to the analysis of large cohort sample sets. Flaherty and co-workers [1] have described a simple method for measuring plasma-bound perfluorooctanoic acid (PFOA) using a 96-well format in three steps: (i) protein precipitation carried out in an Argonaut protein precipitation column, arrayed in a 96-well plate format (Isolute, Argonaut), followed by (ii) repeated vacuum cycles using the extraction plate manifold to draw the crash solvent through the column and then, (iii) the eluate from each column were transferred to autosampler vials [1]. Here, we present an updated and simplified single plate method that employs a methanol protein crash in a PFAS-free 96-well collection plate, followed by centrifugation of the plate, and then subsequent analysis of PFAS from the plate directly by the UHPLC-MS/MS autosampler. The method was demonstrated monitoring the presence and concentrations of 51 PFAS using isotope dilution. The presence of PFAS artifacts (e.g., background) was also examined via extraction blanks. Extraction efficiency and reproducibility were investigated using an *in-house* plasma pool and several blood-based NIST SRMs. To test accuracy and validate the method, we compared our experimentally-derived PFAS values from SRM 1950 to those listed on the certificate of analysis, which has reference values for six PFAS.

### Stock solutions and calibration curve for quantitation

A mixture of 51 non-labeled and 23 mass-labeled PFAS were gravimetrically prepared in methanol and stored at -20 °C. A list of all non-labeled and mass-labeled PFAS species can be found in Supplementary Material Table S1 (along with abbreviations). Calibration curve solutions were prepared by successive dilution from three primary stock solutions to generate a total of 10 levels, ranging from ~ 0.035 to 16 ng.mL^−1^, with equal amounts of spiked internal standards (IS) at ~ 0.80 ng.mL^−1^. Table S2 in the Supplementary Material summarizes the respective concentrations in each level of the calibration curve for non-labeled and mass-labeled PFAS.

### 96-well plate extraction methodology and recovery studies

Optima-grade methanol was used as the protein crash solvent for the method since this solvent produced the fewest PFAS artifacts (data not shown). The volume of the crash solvent was 400 µL methanol per 100 µL of sample (4:1, solvent:sample). All 96-well plates used were rinsed with Optima-grade methanol three times prior to all experiments. The overall extraction methodology, as shown in Graphical Abstract, consisted of (1) adding 100 µL of plasma/serum to a 96-well collection plate, followed by the addition of internal standard (20 µL), (2) samples were then mixed gently in the plate for 5 min at 400 rpm using a Fisher microplate vortexer, (3) 380 µL of methanol were added and vortexed for 5 min at 450 rpm, (4) the well plate was then centrifuged for 15 min at 400 xg, followed by the placement of a micromat onto the plate, and (5) the entire plate was then directly seated in the Vanquish UHPLC autosampler and the injection depth was set to 5 mm to allow only the supernatant layer to be directly sampled by the mass spectrometer.

For the extraction efficiency experiment, 100 µL of the *in-house* plasma pool were added to six different wells on a 96-well collection plate, to which three wells were spiked with 20 µL of IS mixture either pre-extraction (*n* = 3) or post-extraction (*n* = 3), for an assessment of extraction recovery. To the pre-extraction spiked plasma samples, 380 µL of methanol were added (400 µL of methanol was added to the post-extraction spiked samples, making the final volume 500 µL for all samples). The extraction efficiency was also evaluated in water using the same pre- and post-IS spike approach as noted above, except 100 µL of water were used instead of the plasma pool. The results showed similar recoveries for water (on average, approximately 94%) and the *in-house* plasma pool (on average, approximately 82%). Table 1 displays the extraction and method efficiency using methanol.

**Table 1:** Extraction efficiency using the 96-well extraction method described in this manuscript.

Internal standard#	Water	Plasma pool
Recovery *(%)*		
M4PFBA	95.33	81.88
M5PFPeA	93.77	86.44
M3PFBS	92.94	88.98
M2-4:2FTS	88.17	80.98
M5PFHxA	94.68	84.57
M3HFPO-DA	89.35	91.92
M3PFHxS	95.29	81.64
M4PFHpA	94.60	82.69
M2-6:2FTS	100.11	70.07
M8PFOA	95.80	84.77
M8PFOS	91.53	80.17
M9PFNA	95.26	83.51
MFOEA	95.38	85.35
M8FOSA-I	95.42	80.57
M6PFDA	94.43	82.47
d3-N-MeFOSAA	90.47	80.19
M7PFUdA	95.75	81.12
d5-N-EtFOSAA	90.89	77.62
d-N-MeFOSA-M	93.21	82.56
MPFDoA	94.85	80.61
d-N-EtFOSA-M	96.55	82.17
M2PFTeDA	95.05	81.08

# a full list of abbreviations can be found in supplementary material **Table S1**. Plasma pool samples used were an *in-house* plasma pool.

### UHPLC-MS/MS analysis

PFAS analyses were executed using a Thermo Scientific Vanquish UHPLC coupled to a TSQ Quantis triple quadrupole mass spectrometer. Chromatographic separation was achieved using a Gemini C18 column (100 mm x 2 mm; 3 µm) from Phenomenex (Torrence, CA, USA). The UHPLC was fitted with a Vanquish PFAS Replacement Kit, among which included an Acclaim™ 120 C18 (2.1 × 50 mm, 5 µm, 120 Å) as a delay column and UHPLC PFAS-free plumbing and hardware to minimize PFAS background. Water [A] and methanol [B] both containing 5 mM ammonium acetate, were used as the mobile phases. The gradient elution was set as follows: 0–3 min 10% B, 3–4.5 min 10–35% B, 4.5–12.5 min 35–95% B, 12.5–12.51 min 95–99% B, 12.51–19 min 99% and then equilibrated back to initial conditions in 30 min. The autosampler temperature was set to 4 °C and the flow rate and injection volume were set to 0.5 mL.min^−1^ and 10 µL, respectively. Selected-reaction monitoring (SRM) transitions were used to detect and quantify PFAS, with the most intense transition used to quantify the compounds while the second transition was used to confirm identification (if applicable). Table S3 shows all PFAS transitions and additional parameters used for these experiments. Source parameters (in negative mode) were set as follows: ion spray voltage −1500 V and sheath and auxiliary gas set to 50 and 10 arb, respectively. Ion transfer tube temperature was set at 250 °C while the vaporizer temperature was set to 550 °C. Data acquisition and peak integration were performed using Xcalibur v.4.1 software (Thermo Fisher Scientific).

### Data analysis

Quantitation of each detected PFAS was accomplished by integrating the peak related to the most intense transition (quantifier). A total sum of isomers is presented for perfluorohexanesulfonic acid (PFHxS) and perfluorooctanesulfonic acid (PFOS), as ΣPFHxS and ΣPFOS, respectively, as these compounds were monitored as isomeric mixtures. The method only has 23 mass-labeled PFAS, thus for those PFAS which do not have a respective mass-labeled analog, we used the closest IS, selected by retention time, for quantitation purposes. A linear regression model was used to build the calibration equation for each compound and the intercept, slope, and correlation coefficient R^2^ were calculated, along with detection and quantitation limits (LOD and LOQ, respectively). The LOD and LOQ were calculated visually for each compound using signal-to-noise (S/N), (S/N of 3x and 10x for LOD and LOQ, respectively, all with <20% relative standard deviation (RSD)) in replicates of seven. Extraction recovery was investigated by comparison of three replicates of samples spiked before and after extraction (as a %). Precision and accuracy were estimated by analyzing three replicates of a low and mid-level QC (spiked water) and SRMs.

### Background contamination and extraction efficiency

PFAS originating from solvents, labware, hardware and instrumentation has been previously noted, with efforts aimed at both minimizing (e.g., PFAS replacement kits) and validating their presence (e.g., implementation of a variety of blanks). Here, background PFAS levels were investigated in the 96-well plate through blank extractions, which employed Optima grade water instead of plasma (no IS spike). More specifically, three wells were designated blanks and were filled with 100 µL water and 400 µL of methanol to allow for the identification of potential PFAS contamination in the overall extraction workflow. The blank extraction resulted in the detection of 6:2 Fluorotelomer sulfonic acid (6:2 FTS). As a result, 6:2 FTS was excluded from subsequent quantitative analyses.

## Method validation

Calibration curves (performed in the 96-well plates by adding 200 µL of each level) for all 51 PFAS are summarized in Table S4 and include analyte concentration ranges, regression equations, R^2^ values and limits of detection (LOD) and quantitation (LOQ). The coefficient of determination (R^2^) was greater than 0.99 for all analytes with the exception of FDEA and HFDO-PA (0.96 and 0.98, respectively). All calibration curves were linear over the ranges shown. Method selectivity was assessed using an *in-house* plasma pool, no interference was observed for either the analytes or the internal standards. Finally, matrix effect was measured by calculating the ratio of the peak area of the IS in plasma and water samples post extraction. No significant matrix interference was observed, as internal standard peak area ratios ranged from 84 to 103% (data not shown).

To assess method accuracy, QC samples (water spiked with non-labeled PFAS) were analyzed in triplicate at low (0.08 ng.mL^−1^) and middle (0.8 ng.mL^−1^) concentration levels over the course of 3 days (internal standard solution was added for quantitation). Accuracy was calculated at approximately 81% at the low level and 78% at the mid-concentration level, on average, for all PFAS detected (Table S5).

Method accuracy was determined analyzing a commercially-available plasma-based matrix in triplicate (100 µL per replicate), specifically, SRM 1950 – Metabolites in Frozen Human Plasma. Experimentally-derived PFAS concentrations in SRM 1950, using our described method, were compared to the reference values provided in the certificate of analysis for this SRM (Table 2). On average, our percent error results were within acceptable ranges of the values (± 20%) as reported by NIST. For example, PFOA showed −14% in accordance with NIST, PFNA 13%, PFUnA 20%, ΣPFHxS 16% and ΣPFOS 15% but not for PFDA, which was measured at significantly higher levels using our methods (85% higher than for NIST values). Precision (relative standard deviation of triplicate analysis) was calculated and showed values below 5%.

**Table 2:** Comparison between experimentally-derived and reference values noted for standard reference material (SRM) 1950.

Analyte#	NIST*	96-Well plate	Accuracy	Precision
	*Concentration (*ng.mL^−1^*)*		***%***	
PFOA	3.27 ± 0.06	2.81 ± 0.17	14.07	3.78
PFNA	0.72 ± 0.03	0.81 ± 0.02	12.78	2.59
PFDA	0.32 ± 0.01	0.60 ± 0.01	85.40	1.34
PFUnA	0.19 ± 0.01	0.22 ± 0.01	19.89	3.28
PFHxS	3.25 ± 0.08	3.77 ± 0.11	16.09	2.78
ΣPFOS	10.64 ± 0.13	12.24 ± 0.47	15.04	4.12

# a full list of abbreviations can be found in supplementary material Table S1. * reference values by the National Institute of Standards and Technology (NIST) for Standard Reference Material (SRM) 1950 – Metabolites in Frozen Human Plasma can be found at https://www-s.nist.gov/srmors/certificates/1950.pdf. The values provided by our SRM 1950 samples were obtained in a 96-well collection plate using methanol.

Twenty-four (out of 51 monitored) PFAS, of differing chain lengths and chemical moieties, were detected using the described workflow both in healthy human plasma (SRM 1950) and female and male serum (SRM 971) (Table 3). In all analyzed samples, PFAS were determined in a range of 0.05 ng.mL^−1^ to 16.71 ng.mL^−1^. PFBA, PFHxA, ΣPFHxS, ΣPFOS, PFOA, PFNA, 8:2FTS, PFDA and PFHxDA were detected in all samples analyzed. The PFAS with the highest concentration observed was PFOS (Table 3). For the carboxylic acid containing PFAS, PFOA had the highest concentration, with the greatest amount in SRM_971_Male (4.216 ng.mL^−1^). Male serum (971) also had the highest total PFAS when compared to the other materials, with a ΣPFAS of 31.36 ± 0.95 ng mL^−1^. This study has expanded the number of PFAS that can be monitored using SRM 1950, as well as highlighting the first PFAS values for SRM 971, both providing the potential to improve community-wide data harmonization efforts.

**Table 3:** Concentration of PFAS detected in standard reference materials (SRM) 1950 (metabolites in frozen human plasma), and 971 (hormones in frozen human serum, male and female).

Analyte^#^	SRM 1950 Healthy plasma	SRM 971 Female serum	SRM 971 Male serum
*Concentration (*ng.mL^−1^*)*			
PFBA	0.20 ± 0.06	0.19 ± 0.06	0.20 ± 0.01
PFPeA	1.60 ± 0.04	1.63 ± 0.13	1.61 ± 0.04
PFBS	n.d.	n.d.	0.78 ± 0.01
PFHxA	0.36 ± 0.01	0.37 ± 0.00	0.38 ± 0.01
PFPeS	<LOQ	<LOQ	<LOQ
ΣPFHxS	3.77 ± 0.10	2.35 ± 0.17	2.79 ± 0.08
PFHpA	0.24 ± 0.01	0.26 ± 0.01	0.24 ± 0.01
PFECHS	0.36 ± 0.02	0.27 ± 0.00	0.27 ± 0.00
PFHpS	0.42 ± 0.04	0.26 ± 0.02	0.28 ± 0.01
PFOA	2.81 ± 0.17	3.70 ± 0.16	4.22 ± 0.30
ΣPFOS	11.90 ± 0.49	14.21 ± 0.39	16.71 ± 0.55
PFNA	0.81 ± 0.02	1.04 ± 0.06	1.25 ± 0.11
9Cl-PF3ONS	0.15 ± 0.00	0.15 ± 0.00	0.14 ± 0.00
FOSAA	0.15 ± 0.01	0.21 ± 0.02	0.19 ± 0.02
8:2FTS	0.11 ± 0.00	0.13 ± 0.02	0.12 ± 0.01
PFDA	0.60 ± 0.01	0.61 ± 0.01	0.63 ± 0.01
N-MeFOSAA	0.23 ± 0.00	0.53 ± 0.01	0.41 ± 0.04
PFUdA	0.22 ± 0.01	0.29 ± 0.01	0.32 ± 0.01
N-EtFOSAA	n.d.	0.10 ± 0.02	0.06 ± 0.03
PFDoA	<LOQ	<LOQ	<LOQ
6:2diPAP	0.17 ± 0.07	0.41 ± 0.09	0.26 ± 0.06
PFTeDA	0.06 ± 0.00	0.06 ± 0.01	0.06 ± 0.00
PFHxDA	<LOQ	<LOQ	<LOQ
8:2diPAP	n.d.	0.43 ± 0.02	0.41 ± 0.02
**ΣPFAS**	**24.16** **±** **0.63**	**27.22** **±** **1.01**	**31.36** **±** **0.95**

Values are shown as means ± standard deviation in ng.mL^−1^. **^#^** a full list of abbreviations can be found in supplementary material **Table S1**. n.d., not detected; <LOQ, below limits of quantitation.

## Conclusion

The method previously developed by Flaherty and co-workers [1] for quantification of PFOA in serum and plasma samples by direct analysis was adapted, improved and simplified for use with a single 96-well collection plate. These adaptations included the removal of the Isolute 96-well plate and substitution for protein precipitation in a simple 96-well plate reservoir. These improvements allowed us to reduce overall cost, as well as the necessity of an additional 96-well plate as a collection reservoir. Furthermore, the methodology was extended for the identification and quantitation of over 50 PFAS. This simple, quantitatively robust, and relatively background-free method provided comparable results to reference values noted for NIST SRM 1950. Therefore, this work enables high-throughput PFAS profiling from plasma or serum, which may be adopted in large-scale studies at a fraction of the resources of traditional methods (and perhaps even further with the inclusion of robotic platforms). Further work remains in validating this technology for the analysis of other biofluids, as well as continuing to reduce to lower plasma volumes.

## Declaration of Competing Interest

The authors declare that they have no known competing financial interests or personal relationships that could have appeared to influence the work reported in this paper.

## Appendix Supplementary materials

Image, application 1
