# Supplementary material for: A rapid and simple method to quantify per- and polyfluoroalkyl substances (PFAS) in plasma and serum using 96-well plates
Source: MethodsX. 2020 Oct 17;7:101111. doi: 10.1016/j.mex.2020.101111 (PMC7588704; doi:10.1016/j.mex.2020.101111)
Supplement: Supplementary file 1 [file mmc1.docx]

**A rapid and simple method to quantify per- and polyfluoroalkyl substances (PFAS) in plasma and serum using 96-well plates**

Bianca Ferreira Da Silva^1^, Atiye Ahmadireskety^2^, Juan J. Aristizabal-Henao^1^, John A. Bowden^1^

1 College of Veterinary Medicine, Department of Physiological Sciences, University of Florida, Gainesville, Florida, USA

2 Department of Chemistry, University of Florida, Gainesville, Florida, USA

**Corresponding Author:**

Dr. John A. Bowden, Ph.D.

Assistant Professor

Department of Physiological Sciences

College of Veterinary Medicine

University of Florida

1333 Center Drive

Gainesville, FL, USA, 32610

Email: [john.bowden@ufl.edu](mailto:john.bowden@ufl.edu)

Table S1. Abbreviations of all non-labeled and mass-labeled PFAS.

| **Abbreviation** | **Compound** |
| --- | --- |
| M4PFBA | M4-perfluorobutanoic acid |
| M5PFPeA | M5-perfluoropentanoic acid |
| M5PFHxA | M5-perfluorohexanoic acid |
| M4PFHpA | M4-perfluoroheptanoic acid |
| M8PFOA | M8-perfluorooctanoic acid |
| M9PFNA | M9-perfluorononanoic acid |
| M6PFDA | M6-perfluorodecanoic acid |
| M7PFUdA | M7-perfluoroundecanoic acid |
| MPFDoA | M-perfluorododecanoic acid |
| M2PFTeDA | M2-perfluorotetradecanoic acid |
| M8FOSA | M8-perfluorooctane sulfonamide |
| d3-N-MeFOSAA | d3-n-methylperfluoro-1-octanesulfonamidoacetic acid |
| d5-N-EfFOSAA | d5-n-ethylperfluoro-1-octanesulfonamidoacetic acid |
| M3PFBS | M3-perfluorobutyl sulfonate |
| M3PFHxS | M3-perfluorohexyl sulfonate |
| M8PFOS | M8-perfluorooctyl sulfonate |
| M2-4:2FTS | M2-4:2 fluorotelomer sulfonate |
| M2-6:2FTS | M2-6:2 fluorotelomer sulfonate |
| M2-8:2FTS | M2-8:2 fluorotelomer sulfonate |
| MFHEA | M-6:2 fluorotelomer carboxylic acid |
| MFOEA | M-n-2-perfluorooctyl ethanoic acid |
| MFDEA | M-n-2-perfluorodecyl ethanoic acid |
| d3-N-MeFOSA-M | d3-n-methylperfluorooctane-1-sulfonamide |
| M3HFPO-DA | M3-hexafluoropropylene oxide dimer acid (Gen-X) |
| d5-N-EtFOSA-M | d5-n-ethylperfluorooctane-1-sulfonamide |
| PFBA | perfluorobutanoic acid |
| PFPrS | perfluoropropane sulfonate |
| PFPeA | perfluoropentanoic acid |
| PFBS | perfluorobutyl sulfonate |
| FBSA | perfluorobutane sulfonamide |
| 4:2FTS | 4:2 fluorotelomer sulfonate |
| PFHxA | perfluorohexanoic acid |
| PFPeS | perfluoropentane sulfonate |
| HFPO-DA | hexafluoropropylene oxide dimer acid (Gen-X) |
| ΣPFHxS | perfluorohexyl sulfonate |
| PFHpA | perfluoroheptanoic acid |
| NaDONA | dodecafluoro-3H-4,8-dioxanonanoate |
| FHEA | 6:2 fluorotelomer carboxylic acid |
| AP-FHxSA | n-(3-dimethylaminopropan-1-yl)perfluoro-1-hexanesulfonamide |
| PFECHS | n-decafluoro-4 ethylcyclohexanesulfonate |
| FHxSA | perfluorohexane sulfonamide |
| 6:2FTS | 6:2 fluorotelomer sulfonate |
| PFHpS | perfluoroheptane sulfonate |
| PFOA | perfluorooctanoic acid |
| ΣPFOS | perfluorooctyl sulfonate |
| PFNA | perfluorononanoic acid |
| Cl-PFOS | 8-chlorohexadecafluoro-3-oxaoctane-1-sulfonate |
| FOEA | n-2-perfluorooctyl ethanoic acid |
| FOUEA | 2H-perfluoro-2-decanoic acid |
| Cl-PF3ONS | 9-chlorohexadecafluoro-3-oxanonane-1-sulfonate |
| FOSAA | 2-perfluorooctanesulfonamido acetic acid |
| PFNS | perfluorononane sulfonate |
| 8:2FTS | 8:2 fluorotelomer sulfonate |
| FOSA | perfluoro-1-octanesulfonamide |
| PFDA | Perfluorodecanoic acid |
| N-MeFOSAA | N-methylperfluoro-1-octanesulfonamidoacetic acid |
| PFDS | perfluorodecane sulfonate |
| PFUnDA | perfluoroundecanoic acid |
| N-EtFOSAA | N-ethylperfluoro-1-octanesulfonamidoacetic acid |
| FDUEA | n-2-perfluorodecyl ethanoic acid |
| FDEA | n-2-perfluorodecyl ethanoic acid |
| Cl-PF3OUdS | 11-chloroeicosafluoro-3-oxaundecane-1-sulfonate |
| N-MeFOSA | N-methylperfluorooctane sulfonamide |
| PFDoDA | perfluorododecanoic acid |
| 10:2FTS | 10:2 fluorotelomer sulfonate |
| 6:6PFPi | sodium bis(perfluorohexyl)phosphinate |
| N-EtFOSA | N-ethylperfluorooctane sulfonamide |
| PFDoDS | perfluorododecane sulfonate |
| PFTriDA | perfluorotridecanoic acid |
| 6:2diPAP | 6:2 fluorotelomer phosphate diester |
| PFTreDA | perfluorotetradecanoic acid |
| 6:8PFPi | sodium perfluorohexylperfluorooctylphosphinate |
| 6:2/8:2diPAP | (1H,1H,2H,2H-perfluorooctyl-1H,1H,2H,2H-perfluorodecyl)phosphate |
| PFHxDA | perfluorohexadecanoic acid |
| 8:2diPAP | 8:2 fluorotelomer phosphate diester |
| PFODA | perfluorooctadecanoic acid |
| diSAmPAP | sodium bis-(2-N-ethylperfluorooctane-1-sulfonamido)ethyl |
| PFSA | Perfluorosulfonic acids |
| PFCA | Perfluorocarboxylic acids |

**Table S2. Individual concentrations for all levels of the calibration curve for non-labeled and mass-labeled standards.**

| **Analyte^#^** | **Concentration (ng.mL^-1^)** | | | | | | | | | |
| --- | --- | --- | --- | --- | --- | --- | --- | --- | --- | --- |
|  | **Cal01** | **Cal02** | **Cal03** | **Cal04** | **Cal05** | **Cal06** | **Cal07** | **Cal08** | **Cal09** | **Cal10** |
| **Non-Labeled Standards** | | | | | | | | | | |
| PFDoS | 0.032 | 0.072 | 0.149 | 0.355 | 0.752 | 1.495 | 3.551 | 7.523 | 11.437 | 15.492 |
| PFECHS | 0.031 | 0.070 | 0.144 | 0.344 | 0.728 | 1.448 | 3.439 | 7.288 | 11.079 | 15.007 |
| 9Cl-PF3ONS | 0.031 | 0.069 | 0.144 | 0.342 | 0.724 | 1.440 | 3.421 | 7.249 | 11.021 | 14.928 |
| PFODA | 0.033 | 0.074 | 0.153 | 0.365 | 0.771 | 1.533 | 3.642 | 7.717 | 11.731 | 15.890 |
| PFPrS | 0.031 | 0.070 | 0.145 | 0.345 | 0.729 | 1.449 | 3.442 | 7.294 | 11.089 | 15.020 |
| 10:2 FTS | 0.032 | 0.073 | 0.151 | 0.360 | 0.761 | 1.514 | 3.596 | 7.620 | 11.584 | 15.691 |
| 8:2diPAP | 0.033 | 0.076 | 0.157 | 0.374 | 0.790 | 1.571 | 3.732 | 7.907 | 12.021 | 16.283 |
| 6:6 PFPi | 0.032 | 0.072 | 0.150 | 0.358 | 0.756 | 1.504 | 3.572 | 7.569 | 11.507 | 15.586 |
| FBSA-I | 0.036 | 0.081 | 0.167 | 0.398 | 0.842 | 1.675 | 3.979 | 8.431 | 12.817 | 17.361 |
| FHxSA-I | 0.036 | 0.082 | 0.170 | 0.405 | 0.856 | 1.703 | 4.044 | 8.569 | 13.027 | 17.646 |
| N-AP-FHxSA | 0.032 | 0.074 | 0.152 | 0.363 | 0.767 | 1.525 | 3.623 | 7.677 | 11.670 | 15.808 |
| 8Cl-PFOS | 0.032 | 0.072 | 0.149 | 0.354 | 0.749 | 1.489 | 3.536 | 7.493 | 11.391 | 15.429 |
| 6:8 PFPi | 0.033 | 0.075 | 0.155 | 0.369 | 0.780 | 1.552 | 3.687 | 7.812 | 11.876 | 16.087 |
| N-MeFOSA-M | 0.033 | 0.075 | 0.156 | 0.372 | 0.786 | 1.563 | 3.713 | 7.867 | 11.961 | 16.201 |
| N-EtFOSA-M | 0.032 | 0.073 | 0.152 | 0.362 | 0.765 | 1.521 | 3.613 | 7.655 | 11.638 | 15.764 |
| FDEA | 0.036 | 0.081 | 0.167 | 0.398 | 0.842 | 1.675 | 3.979 | 8.431 | 12.817 | 17.361 |
| FOUEA | 0.030 | 0.067 | 0.138 | 0.330 | 0.697 | 1.386 | 3.292 | 6.975 | 10.604 | 14.363 |
| diSAmPAP | 0.029 | 0.066 | 0.137 | 0.325 | 0.688 | 1.368 | 3.250 | 6.886 | 10.468 | 14.179 |
| FOSAA | 0.033 | 0.074 | 0.154 | 0.366 | 0.775 | 1.541 | 3.659 | 7.754 | 11.787 | 15.966 |
| HFPO-DA | 0.075 | 0.170 | 0.352 | 0.838 | 1.772 | 3.523 | 8.369 | 17.733 | 26.958 | 36.516 |
| 11Cl-PF3OUdS | 0.031 | 0.071 | 0.148 | 0.352 | 0.744 | 1.479 | 3.513 | 7.443 | 11.315 | 15.327 |
| 6:2diPAP | 0.034 | 0.077 | 0.159 | 0.380 | 0.803 | 1.596 | 3.791 | 8.034 | 12.213 | 16.543 |
| FHEA | 0.035 | 0.080 | 0.166 | 0.395 | 0.836 | 1.662 | 3.948 | 8.366 | 12.719 | 17.228 |
| FOEA | 0.036 | 0.081 | 0.168 | 0.401 | 0.848 | 1.687 | 4.006 | 8.489 | 12.906 | 17.481 |
| PFHxDA | 0.029 | 0.067 | 0.138 | 0.328 | 0.694 | 1.380 | 3.279 | 6.947 | 10.561 | 14.306 |
| NaDONA | 0.034 | 0.077 | 0.159 | 0.379 | 0.802 | 1.594 | 3.787 | 8.024 | 12.199 | 16.524 |
| 6:2/8:2 diPAP | 0.033 | 0.075 | 0.156 | 0.372 | 0.787 | 1.566 | 3.719 | 7.880 | 11.979 | 16.226 |
| PFBA | 0.039 | 0.088 | 0.182 | 0.434 | 0.918 | 1.826 | 4.336 | 9.188 | 13.968 | 18.920 |
| PFPeA | 0.039 | 0.088 | 0.182 | 0.434 | 0.918 | 1.826 | 4.336 | 9.188 | 13.968 | 18.920 |
| PFHxA | 0.039 | 0.088 | 0.182 | 0.434 | 0.918 | 1.826 | 4.336 | 9.188 | 13.968 | 18.920 |
| PFHpA | 0.039 | 0.088 | 0.182 | 0.434 | 0.918 | 1.826 | 4.336 | 9.188 | 13.968 | 18.920 |
| PFOA | 0.039 | 0.088 | 0.182 | 0.434 | 0.918 | 1.826 | 4.336 | 9.188 | 13.968 | 18.920 |
| PFNA | 0.039 | 0.088 | 0.182 | 0.434 | 0.918 | 1.826 | 4.336 | 9.188 | 13.968 | 18.920 |
| PFDA | 0.039 | 0.088 | 0.182 | 0.434 | 0.918 | 1.826 | 4.336 | 9.188 | 13.968 | 18.920 |
| PFUdA | 0.039 | 0.088 | 0.182 | 0.434 | 0.918 | 1.826 | 4.336 | 9.188 | 13.968 | 18.920 |
| PFDoA | 0.039 | 0.088 | 0.182 | 0.434 | 0.918 | 1.826 | 4.336 | 9.188 | 13.968 | 18.920 |
| PDTrDA | 0.039 | 0.088 | 0.182 | 0.434 | 0.918 | 1.826 | 4.336 | 9.188 | 13.968 | 18.920 |
| PFTeDA | 0.039 | 0.088 | 0.182 | 0.434 | 0.918 | 1.826 | 4.336 | 9.188 | 13.968 | 18.920 |
| FOSA | 0.039 | 0.088 | 0.182 | 0.434 | 0.918 | 1.826 | 4.336 | 9.188 | 13.968 | 18.920 |
| N-MeFOSAA | 0.039 | 0.088 | 0.182 | 0.434 | 0.918 | 1.826 | 4.336 | 9.188 | 13.968 | 18.920 |
| N-EtFOSAA | 0.039 | 0.088 | 0.182 | 0.434 | 0.918 | 1.826 | 4.336 | 9.188 | 13.968 | 18.920 |
| PFBS | 0.034 | 0.078 | 0.161 | 0.384 | 0.812 | 1.616 | 3.838 | 8.131 | 12.362 | 16.744 |
| PFPeS | 0.037 | 0.083 | 0.171 | 0.408 | 0.863 | 1.716 | 4.076 | 8.637 | 13.130 | 17.785 |
| ΣPFHxS | 0.035 | 0.080 | 0.166 | 0.396 | 0.837 | 1.665 | 3.955 | 8.379 | 12.739 | 17.255 |
| PFhpS | 0.037 | 0.084 | 0.173 | 0.412 | 0.872 | 1.734 | 4.119 | 8.729 | 13.270 | 17.974 |
| ΣPFOS | 0.036 | 0.081 | 0.169 | 0.402 | 0.850 | 1.690 | 4.013 | 8.503 | 12.927 | 17.511 |
| PFNS | 0.037 | 0.084 | 0.175 | 0.417 | 0.881 | 1.753 | 4.163 | 8.820 | 13.409 | 18.163 |
| PFDS | 0.038 | 0.085 | 0.176 | 0.419 | 0.886 | 1.762 | 4.184 | 8.866 | 13.479 | 18.258 |
| 4:2FTS | 0.036 | 0.082 | 0.170 | 0.406 | 0.858 | 1.707 | 4.054 | 8.591 | 13.060 | 17.690 |
| 6:2FTS | 0.037 | 0.084 | 0.173 | 0.412 | 0.872 | 1.734 | 4.119 | 8.729 | 13.270 | 17.974 |
| 8:2FTS | 0.037 | 0.084 | 0.175 | 0.417 | 0.881 | 1.753 | 4.163 | 8.820 | 13.409 | 18.163 |
| **Mass-Labeled Standards** | | | | | | | | | | |
| MPFBA | 0.786 | 0.828 | 0.831 | 0.810 | 0.833 | 0.803 | 0.807 | 0.835 | 0.826 | 0.819 |
| M5PFPeA | 0.786 | 0.828 | 0.831 | 0.810 | 0.833 | 0.803 | 0.807 | 0.835 | 0.826 | 0.819 |
| M5PFHxA | 0.786 | 0.828 | 0.831 | 0.810 | 0.833 | 0.803 | 0.807 | 0.835 | 0.826 | 0.819 |
| M4PFHpA | 0.786 | 0.828 | 0.831 | 0.810 | 0.833 | 0.803 | 0.807 | 0.835 | 0.826 | 0.819 |
| M8PFOA | 0.786 | 0.828 | 0.831 | 0.810 | 0.833 | 0.803 | 0.807 | 0.835 | 0.826 | 0.819 |
| M9PFNA | 0.786 | 0.828 | 0.831 | 0.810 | 0.833 | 0.803 | 0.807 | 0.835 | 0.826 | 0.819 |
| M6PFDA | 0.786 | 0.828 | 0.831 | 0.810 | 0.833 | 0.803 | 0.807 | 0.835 | 0.826 | 0.819 |
| M7PFUdA | 0.786 | 0.828 | 0.831 | 0.810 | 0.833 | 0.803 | 0.807 | 0.835 | 0.826 | 0.819 |
| MPFDoA | 0.786 | 0.828 | 0.831 | 0.810 | 0.833 | 0.803 | 0.807 | 0.835 | 0.826 | 0.819 |
| M2PFTeDA | 0.786 | 0.828 | 0.831 | 0.810 | 0.833 | 0.803 | 0.807 | 0.835 | 0.826 | 0.819 |
| M8FOSA | 0.786 | 0.828 | 0.831 | 0.810 | 0.833 | 0.803 | 0.807 | 0.835 | 0.826 | 0.819 |
| d3-N-MeFOSAA | 0.786 | 0.828 | 0.831 | 0.810 | 0.833 | 0.803 | 0.807 | 0.835 | 0.826 | 0.819 |
| d5-N-EfFOSAA | 0.786 | 0.828 | 0.831 | 0.810 | 0.833 | 0.803 | 0.807 | 0.835 | 0.826 | 0.819 |
| M3PFBS | 0.730 | 0.769 | 0.772 | 0.753 | 0.774 | 0.746 | 0.749 | 0.776 | 0.767 | 0.760 |
| M3PFHxS | 0.743 | 0.783 | 0.786 | 0.767 | 0.788 | 0.760 | 0.763 | 0.790 | 0.781 | 0.774 |
| M8PFOS | 0.752 | 0.792 | 0.795 | 0.776 | 0.797 | 0.769 | 0.772 | 0.799 | 0.790 | 0.783 |
| M2-4:2FTS | 0.735 | 0.774 | 0.777 | 0.758 | 0.779 | 0.751 | 0.754 | 0.781 | 0.772 | 0.765 |
| M2-6:2FTS | 0.746 | 0.785 | 0.789 | 0.769 | 0.790 | 0.762 | 0.766 | 0.792 | 0.784 | 0.777 |
| M2-8:2FTS | 0.753 | 0.793 | 0.796 | 0.776 | 0.798 | 0.769 | 0.773 | 0.800 | 0.791 | 0.784 |
| MFOEA | 0.817 | 0.861 | 0.864 | 0.843 | 0.866 | 0.835 | 0.839 | 0.868 | 0.859 | 0.851 |
| d3-N-MeFOSA-M | 4.197 | 4.421 | 4.438 | 4.329 | 4.448 | 4.291 | 4.309 | 4.460 | 4.411 | 4.372 |
| M3HFPO-DA | 3.945 | 4.154 | 4.171 | 4.068 | 4.180 | 4.032 | 4.049 | 4.191 | 4.145 | 4.109 |
| d5-N-EtFOSA-M | 3.957 | 4.168 | 4.184 | 4.081 | 4.193 | 4.045 | 4.062 | 4.204 | 4.158 | 4.122 |

^#^ a full list of abbreviations can be found in supplementary material **Table S1**

**Table S3.** All transition parameters for PFAS analysis Thermo Quantis – SRM experiment set.

| **Analyte^#^** | **Retention Time (min)** | **Window (min)** | **Precursor Ion (m/z)** | **Fragment Ion (m/z)** | **Collision Energy (V)** | **Dwell Time (ms)** | **RF Lens (V)** |
| --- | --- | --- | --- | --- | --- | --- | --- |
| PFBA | 4.97 | 1 | 213.0 | 168.9 | 11 | 47.97 | 75 |
| M4PFBA | 4.97 | 1 | 217.0 | 172.0 | 11 | 47.97 | 75 |
| PFPrS1 | 5.37 | 1 | 249.1 | 80.0 | 29 | 25.81 | 138 |
| PFPrS2 | 5.37 | 1 | 249.1 | 98.9 | 26 | 25.81 | 138 |
| PFPeA | 5.69 | 1 | 263.0 | 219.0 | 11 | 20.61 | 64 |
| M5PFPeA | 5.69 | 1 | 268.0 | 223.0 | 11 | 20.61 | 64 |
| PFBS1 | 5.74 | 1 | 299.0 | 80.0 | 32 | 20.61 | 152 |
| PFBS2 | 5.74 | 1 | 299.0 | 99.0 | 28 | 20.61 | 152 |
| M3PFBS | 5.75 | 1 | 302.0 | 99.0 | 29 | 20.61 | 152 |
| 4:2FTS2 | 6.05 | 1 | 327.0 | 81.0 | 29 | 20.61 | 138 |
| 4:2FTS1 | 6.05 | 1 | 327.0 | 306.9 | 18 | 20.61 | 138 |
| M2-4:2FTS | 6.05 | 1 | 329.0 | 81.0 | 18 | 20.61 | 138 |
| FBSA-1 | 6.06 | 1 | 298.0 | 78.0 | 25 | 20.61 | 144 |
| FBSA-2 | 6.06 | 1 | 298.0 | 119.0 | 18 | 20.61 | 144 |
| PFHxA | 6.08 | 1 | 312.9 | 269.0 | 10 | 20.61 | 73 |
| M5PFHxA | 6.08 | 1 | 318.0 | 273.0 | 10 | 20.61 | 73 |
| PFPeS1 | 6.1 | 1 | 349.0 | 80.0 | 34 | 20.61 | 174 |
| PFPeS2 | 6.1 | 1 | 349.0 | 99.0 | 31 | 20.61 | 174 |
| HFPO-DA | 6.17 | 1 | 329.0 | 285.0 | 7 | 19.50 | 65 |
| M3HFPO-DA | 6.22 | 1 | 332.0 | 287.0 | 7 | 17.73 | 65 |
| NaDONA | 6.51 | 1 | 377.0 | 251.0 | 10 | 17.73 | 280 |
| M4PFHpA | 6.57 | 1 | 367.0 | 322.0 | 11 | 17.73 | 81 |
| ΣPFHxS2 | 6.57 | 1 | 399.0 | 80.0 | 37 | 17.73 | 174 |
| M3PFHxS | 6.57 | 1 | 402.0 | 99.0 | 35 | 17.73 | 151 |
| N-AP-FHxSA-2 | 7.07 | 2 | 483.1 | 318.9 | 22 | 14.47 | 244 |
| ΣPFHxS1 | 6.59 | 1 | 399.0 | 99.0 | 35 | 17.73 | 174 |
| M2-6:2FTS1 | 7.28 | 2 | 429.0 | 376.0 | 21 | 14.04 | 166 |
| FHEA | 6.86 | 1 | 376.9 | 293.0 | 20 | 15.01 | 100 |
| PFHpA2 | 6.95 | 1 | 362.9 | 318.9 | 11 | 15.01 | 81 |
| PFHpA1 | 6.98 | 1 | 362.9 | 169.0 | 17 | 14.47 | 81 |
| PFECHS2 | 7.07 | 1 | 460.9 | 99.0 | 29 | 14.47 | 171 |
| PFECHS1 | 7.07 | 1 | 460.9 | 381.0 | 26 | 14.47 | 171 |
| N-AP-FHxSA-1 | 7.08 | 1 | 483.1 | 169.0 | 27 | 14.47 | 244 |
| 6:2FTS2 | 7.12 | 1 | 427.0 | 81.0 | 30 | 14.47 | 166 |
| 6:2FTS1 | 7.12 | 1 | 427.0 | 406.9 | 21 | 14.47 | 166 |
| M2-6:2FTS2 | 7.12 | 1 | 429.0 | 81.0 | 21 | 14.47 | 166 |
| PFHpS2 | 7.14 | 1 | 448.9 | 98.9 | 38 | 14.47 | 223 |
| PFOA2 | 7.16 | 1 | 413.0 | 369.0 | 11 | 14.47 | 94 |
| M8PFOA | 7.16 | 1 | 421.0 | 376.0 | 11 | 14.47 | 94 |
| PFHpS1 | 7.16 | 1 | 448.9 | 80.0 | 39 | 14.47 | 223 |
| FHxSA-2 | 7.18 | 1 | 398.0 | 378.0 | 20 | 14.47 | 185 |
| PFOA1 | 7.18 | 1 | 413.0 | 169.0 | 19 | 14.47 | 94 |
| FHxSA-1 | 7.19 | 1 | 398.0 | 78.0 | 28 | 14.47 | 185 |
| FOEA | 8.28 | 2 | 476.9 | 393.0 | 18 | 14.04 | 100 |
| MFOEA-2 | 8.3 | 2 | 479.0 | 435.0 | 11 | 14.04 | 107 |
| M8PFOS1 | 7.82 | 1 | 507.0 | 80.0 | 44 | 14.04 | 259 |
| ΣPFOS1 | 7.83 | 1 | 498.9 | 80.0 | 40 | 14.04 | 214 |
| M8PFOS2 | 7.83 | 1 | 507.0 | 99.0 | 44 | 14.04 | 259 |
| M9PFNA | 7.84 | 1 | 472.0 | 427.0 | 12 | 14.04 | 101 |
| ΣPFOS2 | 7.84 | 1 | 498.9 | 99.0 | 41 | 14.04 | 214 |
| PFNA1 | 7.85 | 1 | 463.0 | 219.0 | 16 | 14.04 | 101 |
| PFNA2 | 7.86 | 1 | 463.0 | 419.0 | 12 | 14.04 | 101 |
| 8Cl-PFOS2 | 7.95 | 1 | 514.9 | 98.9 | 40 | 14.04 | 273 |
| 8Cl-PFOS1 | 7.96 | 1 | 514.9 | 80.0 | 41 | 14.04 | 273 |
| FOUEA | 8.07 | 1 | 456.9 | 392.9 | 12 | 14.04 | 100 |
| MFOEA-1 | 8.09 | 1 | 479.0 | 394.0 | 11 | 14.04 | 107 |
| 9Cl-PF3ONS1 | 8.25 | 1 | 530.8 | 351.0 | 25 | 14.04 | 155 |
| PFDA | 8.76 | 2 | 513.0 | 469.0 | 11 | 14.04 | 94 |
| FOSAA1 | 8.48 | 1 | 556.0 | 497.9 | 26 | 14.04 | 210 |
| FOSAA2 | 8.5 | 1 | 556.0 | 419.0 | 23 | 14.04 | 210 |
| 8:2FTS2 | 8.54 | 1 | 527.0 | 486.8 | 31 | 14.04 | 179 |
| 8:2FTS1 | 8.55 | 1 | 527.0 | 506.9 | 25 | 14.04 | 179 |
| PFNS1 | 8.55 | 1 | 549.0 | 80.0 | 43 | 14.04 | 280 |
| PFNS2 | 8.55 | 1 | 549.0 | 99.0 | 43 | 14.04 | 280 |
| M2-8:2FTS | 8.56 | 1 | 529.0 | 81.0 | 25 | 14.04 | 179 |
| MPFDA | 8.57 | 1 | 515.0 | 470.0 | 11 | 14.04 | 94 |
| M6PFDA | 8.57 | 1 | 519.0 | 474.0 | 11 | 14.04 | 94 |
| M8FOSA-I2 | 8.65 | 1 | 506.0 | 78.0 | 30 | 14.04 | 196 |
| M8FOSA-I1 | 8.65 | 1 | 506.0 | 485.9 | 23 | 14.04 | 196 |
| FOSA-I1 | 8.66 | 1 | 498.0 | 78.0 | 30 | 14.04 | 206 |
| FOSA-I2 | 8.76 | 1 | 498.0 | 477.9 | 23 | 14.04 | 206 |
| d3-N-MeFOSAA1 | 8.91 | 1 | 573.0 | 418.9 | 19 | 14.57 | 174 |
| d3-N-MeFOSAA2 | 8.91 | 1 | 573.0 | 482.9 | 14 | 14.57 | 174 |
| N-MeFOSAA1 | 8.92 | 1 | 570.0 | 418.9 | 18 | 14.57 | 178 |
| N-MeFOSAA2 | 8.97 | 1 | 570.0 | 482.9 | 14 | 14.57 | 178 |
| PFDS2 | 9.22 | 1 | 598.9 | 98.9 | 47 | 14.57 | 280 |
| N-EtFOSAA2 | 9.25 | 1 | 584.0 | 419.0 | 19 | 14.57 | 179 |
| N-EtFOSAA1 | 9.25 | 1 | 584.0 | 525.9 | 18 | 14.57 | 179 |
| PFDS1 | 9.25 | 1 | 598.9 | 80.0 | 45 | 14.57 | 280 |
| M7PFUndA2 | 9.27 | 1 | 570.0 | 525.0 | 11 | 14.57 | 116 |
| d5-N-EtFOSAA1 | 9.27 | 1 | 589.0 | 531.0 | 19 | 14.57 | 176 |
| PFUdA | 9.28 | 1 | 563.0 | 519.0 | 11 | 14.57 | 116 |
| d5-N-EtFOSAA2 | 9.28 | 1 | 589.0 | 419.0 | 19 | 14.57 | 176 |
| N-MeFOSA-M2 | 9.92 | 2 | 512.0 | 169.0 | 26 | 14.57 | 187 |
| FDUEA | 9.71 | 1.5 | 557.0 | 493.0 | 21 | 14.57 | 280 |
| 11Cl-PF3OUdS | 9.62 | 1 | 631.0 | 450.9 | 27 | 18.65 | 225 |
| 10:2FTS1 | 10.14 | 2 | 627.0 | 606.9 | 29 | 18.65 | 280 |
| d-N-MeFOSA-M2 | 9.84 | 1 | 515.0 | 169.0 | 26 | 19.48 | 194 |
| d-N-MeFOSA-M1 | 9.84 | 1 | 515.0 | 218.9 | 24 | 19.48 | 194 |
| N-MeFOSA-M1 | 9.87 | 1 | 512.0 | 219.0 | 24 | 19.48 | 187 |
| PFDoA2 | 9.92 | 1 | 612.9 | 319.0 | 18 | 19.48 | 129 |
| PFDoA1 | 9.93 | 1 | 612.9 | 569.0 | 10 | 19.48 | 129 |
| MPFDoA | 9.94 | 1 | 615.0 | 570.0 | 10 | 19.48 | 129 |
| 10:2FTS2 | 9.94 | 1 | 627.0 | 81.0 | 34 | 19.48 | 280 |
| 6:6PFPi | 10.18 | 1 | 700.9 | 400.9 | 55 | 19.48 | 188 |
| d-N-EtFOSA-M1 | 10.29 | 1 | 530.9 | 169.0 | 27 | 19.69 | 203 |
| d-N-EtFOSA-M2 | 10.29 | 1 | 530.9 | 219.0 | 25 | 19.69 | 203 |
| N-EtFOSA-M2 | 10.3 | 1 | 526.0 | 169.0 | 27 | 19.69 | 209 |
| N-EtFOSA-M1 | 10.31 | 1 | 526.0 | 219.0 | 24 | 19.69 | 209 |
| PFDoS2 | 10.45 | 1 | 698.9 | 99.0 | 55 | 19.69 | 280 |
| PFDoS1 | 10.46 | 1 | 698.9 | 80.0 | 55 | 19.69 | 280 |
| PFTrDA1 | 10.53 | 1 | 662.8 | 619.0 | 11 | 19.69 | 136 |
| PFTrDA2 | 10.55 | 1 | 662.8 | 319.0 | 19 | 19.69 | 136 |
| 6:2diPAP2 | 11.06 | 2 | 788.9 | 96.9 | 29 | 19.69 | 216 |
| 6:2diPAP1 | 11.06 | 2 | 788.9 | 442.9 | 17 | 19.69 | 216 |
| PFTeDA | 11.06 | 1 | 713.0 | 669.0 | 12 | 27.42 | 107 |
| M2PFTeDA | 11.06 | 1 | 715.0 | 670.0 | 12 | 27.42 | 107 |
| 6:8PFPi2 | 11.11 | 1 | 800.9 | 400.9 | 55 | 27.42 | 188 |
| 6:8PFPi1 | 11.11 | 1 | 800.9 | 500.8 | 55 | 27.42 | 188 |
| 6:2/8:2diPAP | 11.67 | 1 | 889.0 | 443.0 | 20 | 53.53 | 280 |
| PFHxDA1 | 11.96 | 1 | 812.9 | 768.9 | 12 | 53.53 | 167 |
| PFHxDA2 | 11.97 | 1 | 812.9 | 419.0 | 20 | 53.53 | 167 |
| 8:2diPAP | 12.29 | 1 | 989.0 | 543.0 | 20 | 69.23 | 280 |
| 8:2diPAP2 | 12.31 | 1 | 988.9 | 523.0 | 27 | 69.23 | 280 |
| PFODA1 | 12.69 | 1 | 912.9 | 868.9 | 13 | 80.86 | 188 |
| PFODA2 | 12.7 | 1 | 912.9 | 318.9 | 25 | 80.86 | 188 |
| diSAmPAP | 12.96 | 1 | 1203.0 | 526.0 | 25 | 80.86 | 280 |

-1 and -2: first and second transition of the analyte, whereby the first transition was used for quantitation and the second transition for confirmation. ^#^ a full list of abbreviations can be found in supplementary material **Table S1**.

**Table S4.** Calibration curve ranges, limits of detection and limits of quantitation for each analyte.

|  |  | **Analyte Concentration Range (ng.mL^-1^)** | |  | |  | | |  | | |  |  |  |
| --- | --- | --- | --- | --- | --- | --- | --- | --- | --- | --- | --- | --- | --- | --- |
| **Analyte^#^** | **Internal Standard** | **Min** | **Max** | **Slope** | **Y-intercept** | | **R^2^** | **LOD^a^** | | **LOQ^b^** | **RSD (%)^c^** | | | **Precision (%)^d^** |
| PFBA | IS_M4PFBA | 0.039 | 18.920 | 0.953 | -0.040 | | 0.9998 | 0.004 | | 0.008 | 4.53 | | | 98.53 |
| PFPrS | IS_M5PFPeA | 0.0309 | 15.0201 | 0.378 | -0.039 | | 0.9998 | 0.002 | | 0.006 | 9.31 | | | 101.86 |
| PFPeA | IS_M5PFPeA | 0.0389 | 18.920 | 1.104 | -0.277 | | 0.9994 | 0.021 | | 0.182 | 0.61 | | | 120.26 |
| PFBS | IS_M3PFBS | 0.078 | 16.744 | 1.226 | -0.242 | | 0.9993 | 0.006 | | 0.019 | 7.09 | | | 118.77 |
| FBSA | IS_M3PFBS | 0.1671 | 17.361 | 1.841 | -1.221 | | 0.9990 | 0.081 | | 0.398 | 7.33 | | | 106.13 |
| 4_2FTS | IS_M2-4_2FTS | 0.1703 | 17.6903 | 18.534 | 2.365 | | 0.9986 | 0.007 | | 0.020 | 9.74 | | | 90.77 |
| PFHxA | IS_M5PFHxA | 0.1821 | 18.9201 | 0.983 | -0.086 | | 0.9999 | 0.004 | | 0.021 | 2.99 | | | 116.85 |
| PFPeS | IS_M3HFPO-DA | 0.1712 | 17.785 | 41.448 | -0.618 | | 0.9997 | 0.007 | | 0.020 | 10.55 | | | 114.91 |
| HFPO-DA | IS_M3HFPO-DA | 3.523 | 36.516 | 1.715 | -0.112 | | 0.9890 | 0.838 | | 3.523 | 5.95 | | | 99.37 |
| ΣPFHxS | IS_M3HFPO-DA | 0.1661 | 17.255 | 1.162 | -0.182 | | 0.9995 | 0.019 | | 0.035 | 9.09 | | | 110.10 |
| PFHpA | IS_M4PFHpA | 0.1821 | 18.9201 | 1.042 | -0.027 | | 0.9999 | 0.007 | | 0.021 | 5.21 | | | 110.88 |
| NaDONA | IS_M4PFHpA | 0.802 | 16.524 | 0.007 | -0.004 | | 0.9950 | 0.077 | | 0.379 | 2.27 | | | 87.83 |
| FHEA | IS_M4PFHpA | 0.166 | 17.228 | 0.030 | -0.004 | | 0.9932 | 0.035 | | 0.166 | 0.98 | | | 115.48 |
| N-AP-FHxSA | IS_M2-6_2FTS | 0.767 | 15.808 | 3.288 | -0.797 | | 0.9926 | 0.033 | | 0.363 | 6.14 | | | 85.07 |
| PFECHS | IS_M2-6_2FTS2 | 0.1445 | 15.007 | 141.670 | -9.246 | | 0.9986 | 0.003 | | 0.017 | 8.55 | | | 88.62 |
| FHxSA- | IS_M3PFHxS | 0.070 | 15.007 | 0.212 | 0.040 | | 0.9952 | 0.010 | | 0.036 | 15.06 | | | 99.77 |
| 6_2FTS | IS_M2-6_2FTS | 0.037 | 8.7285 | 18.264 | -0.060 | | 0.9978 | 0.007 | | 0.020 | 15.49 | | | 107.01 |
| PFHpS | IS_M2-6_2FTS | 0.037 | 8.7285 | 9.163 | -0.395 | | 0.9981 | 0.010 | | 0.037 | 13.06 | | | 82.06 |
| PFOA | IS_M8PFOA | 0.0389 | 9.1879 | 1.049 | -0.022 | | 0.9999 | 0.010 | | 0.039 | 4.35 | | | 115.89 |
| ΣPFOS | IS_M8PFOS | 0.0814 | 17.511 | 1.161 | -0.021 | | 0.9998 | 0.019 | | 0.081 | 8.59 | | | 111.77 |
| PFNA | IS_M9PFNA | 0.0389 | 18.9201 | 0.987 | -0.027 | | 0.9999 | 0.007 | | 0.021 | 11.08 | | | 114.19 |
| 8Cl-PFOS | IS_M8PFOS | 0.072 | 11.391 | 0.827 | 0.025 | | 0.9991 | 0.017 | | 0.072 | 2.08 | | | 96.26 |
| FOEA | IS_MFOEA | 0.168 | 17.481 | 0.965 | 0.054 | | 0.9949 | 0.036 | | 0.168 | 5.62 | | | 83.63 |
| FOUEA | IS_MFOEA | 0.030 | 6.975 | 12.705 | -0.717 | | 0.9992 | 0.005 | | 0.006 | 18.82 | | | 110.15 |
| 9Cl-PF3ONS | IS_M2-8_2FTS | 0.0694 | 14.9280 | 206.632 | -7.522 | | 0.9996 | 0.003 | | 0.006 | 6.99 | | | 96.44 |
| FOSAA | IS_M2_8_2FTS | 0.074 | 15.9664 | 47.723 | -1.280 | | 0.9993 | 0.009 | | 0.018 | 12.73 | | | 90.72 |
|  |  | **Analyte Concentration Range (ng.mL^-1^)** | |  | |  | | |  | | |  |  |  |
| **Analyte^#^** | **Internal Standard** | **Min** | **Max** | **Slope** | **Y-intercept** | | **R^2^** | **LOD^a^** | | **LOQ^b^** | **RSD (%)^c^** | | | **Precision (%)^d^** |
| PFNS | IS_M2_8_2FTS | 0.175 | 13.409 | 8.708 | -0.641 | | 0.9990 | 0.010 | | 0.085 | 12.93 | | | 106.97 |
| 8_2FTS | IS_M2-8_2FTS | 0.0373 | 8.8204 | 18.868 | -0.191 | | 0.9996 | 0.020 | | 0.037 | 6.38 | | | 99.46 |
| FOSA-I | IS_M8FOSA-I1 | 0.088 | 9.11879 | 1.123 | -0.092 | | 0.9996 | 0.010 | | 0.039 | 13.38 | | | 89.09 |
| PFDA | IS_M6PFDA | 0.088 | 13.968 | 1.023 | -0.099 | | 0.9999 | 0.004 | | 0.008 | 2.81 | | | 211.65 |
| N-MeFOSAA | IS_d3_N-MeFOSAA1 | 0.088 | 18.9201 | 1.166 | 0.022 | | 0.9995 | 0.008 | | 0.088 | 2.30 | | | 96.92 |
| PFDS | IS_d5-N-EtFOSAA | 0.1758 | 18.2579 | 0.748 | 0.064 | | 0.9992 | 0.020 | | 0.085 | 3.01 | | | 83.77 |
| PFUdA | IS_M7PFUdA | 0.0389 | 18.9201 | 0.965 | -0.016 | | 0.9999 | 0.004 | | 0.021 | 0.89 | | | 111.35 |
| N-EtFOSAA | IS_d5-N-EtFOSAA1 | 0.088 | 18.9201 | 1.168 | 0.004 | | 0.9999 | 0.017 | | 0.073 | 7.02 | | | 113.74 |
| FDEA | IS_M7PFDUdA | 0.167 | 17.361 | 0.016 | 0.021 | | 0.9665 | 0.036 | | 0.167 | 3.05 | | | 96.28 |
| 11Cl-PF3OUdS | IS_d5-N-EtFOSAA | 0.031 | 7.443 | 65.384 | -0.287 | | 0.9995 | 0.003 | | 0.007 | 6.19 | | | 115.77 |
| N-MeFOSA-M | IS_d-N-MeFOSA-M1 | 0.033 | 7.867 | 1.076 | -0.007 | | 0.9999 | 0.006 | | 0.033 | 5.79 | | | 108.34 |
| PFDoA | IS_MPFDoA | 0.039 | 13.9680 | 0.951 | 0.003 | | 0.9998 | 0.004 | | 0.008 | 8.59 | | | 116.94 |
| 10_2FTS | IS_MPFDoA | 0.073 | 11.5841 | 0.074 | 0.001 | | 0.9999 | 0.009 | | 0.032 | 9.33 | | | 106.27 |
| 6_6PFPi | IS_MPFDoA | 0.032 | 7.569 | 0.723 | -0.009 | | 0.9999 | 0.006 | | 0.017 | 7.25 | | | 112.92 |
| N-EtFOSA-M | IS_d-N-EtFOSA-M3 | 0.032 | 7.655 | 1.152 | -0.009 | | 0.9999 | 0.017 | | 0.032 | 3.62 | | | 97.06 |
| PFDoS | IS_MPFDoA | 0.072 | 11.437 | 0.029 | -0.001 | | 0.9992 | 0.017 | | 0.032 | 0.62 | | | 110.54 |
| PFTrDA | IS_d-N-EtFOSA-M3 | 0.9179 | 18.9201 | 0.768 | 0.248 | | 0.9993 | 0.004 | | 0.008 | 4.83 | | | 100.41 |
| 6_2diPAP | IS_M2PFTeDA | 0.034 | 8.034 | 0.083 | -0.002 | | 0.9999 | 0.006 | | 0.018 | 5.18 | | | 101.04 |
| PFTeDA | IS_M2PFTeDA | 0.039 | 13.968 | 0.937 | -0.011 | | 0.9998 | 0.007 | | 0.021 | 1.22 | | | 108.88 |
| 6_8PFPi | IS_M2PFTeDA | 0.033 | 16.0869 | 0.623 | -0.002 | | 0.9999 | 0.002 | | 0.009 | 9.33 | | | 109.97 |
| 6_2_8_2diPAP | IS_M2PFTeDA | 0.033 | 16.2263 | 0.040 | 0.0001 | | 0.9996 | 0.007 | | 0.033 | 4.16 | | | 104.60 |
| PFHxDA | IS_M2PFTeDA | 0.0294 | 10.5614 | 1.416 | 0.002 | | 0.9999 | 0.003 | | 0.006 | 11.95 | | | 99.58 |
| 8_2diPAP | IS_M2PFTeDA | 0.1568 | 16.2834 | 0.056 | -0.0056 | | 0.9992 | 0.009 | | 0.034 | 5.45 | | | 94.09 |
| PFODA | IS_M2PFTeDA | 0.033 | 15.8904 | 1.257 | 0.017 | | 0.9998 | 0.003 | | 0.007 | 9.98 | | | 101.78 |
| diSAmPAP | IS_M2PFTeDA | 0.029 | 6.886 | 0.032 | 0.002 | | 0.9963 | 0.016 | | 0.029 | 5.54 | | | 107.08 |

^#^ a full list of abbreviations can be found in supplementary material **Table S1**. LOD, limit of detection; LOQ, limit of quantitation.

^a^ LOD (S/N > 3), ^b^ LOQ (S/N > 10), ^C^ LOQ bias showed as RSD (%) (n=7), ^d^ LOQ precision (%) (n=7).

**Table S5.** Quality control sample analysis at low- (LQC) and mid- (MQC) concentration levels.

| **Analyte^#^** | **LQC** | **MQC** |
| --- | --- | --- |
|  | *Accuracy (%)* | |
| PFBA | 66.18 | 77.98 |
| PFPrS | 96.20 | 75.16 |
| PFPeA | 79.98 | 74.56 |
| PFBS | 73.44 | 74.14 |
| FBSA | 66.20 | 71.21 |
| 4:2FTS | 93.02 | 79.51 |
| PFHxA | 81.73 | 77.02 |
| PFPeS | 87.61 | 79.45 |
| HFPO-DA* | n.d. | 93.36 |
| ΣPFHxS | 72.12 | 69.90 |
| PFHpA | 87.19 | 76.99 |
| NaDONA | 93.81 | 86.20 |
| FHEA* | n.d. | 66.04 |
| N-AP-FHxSA | 76.67 | 68.57 |
| PFECHS | 77.32 | 72.80 |
| FHxSA | 68.77 | 74.46 |
| PFHpS | 64.04 | 67.98 |
| PFOA | 82.88 | 76.15 |
| ΣPFOS | 94.62 | 72.64 |
| PFNA | 82.33 | 76.25 |
| 8Cl-PFOS | 94.73 | 79.13 |
| FOUEA | 75.84 | 76.92 |
| FOEA | 92.87 | 95.42 |
| 9Cl-PF3ONS | 78.72 | 73.98 |
| FOSAA | 84.88 | 74.54 |
| PFNS | 79.89 | 76.51 |
| FOSA-I | 70.14 | 81.04 |
| 8:2FTS | 64.19 | 77.40 |
| PFDA | 84.37 | 77.58 |
| N-MeFOSAA | 90.76 | 77.62 |
| PFDS | 83.81 | 78.51 |
| PFUdA | 81.67 | 76.82 |
| N-EtFOSAA | 77.16 | 85.77 |
| FDEA | 67.05 | 93.83 |
| 11Cl-PF3OUdS | 77.52 | 70.52 |
| N-MeFOSA-M | 85.16 | 83.06 |
| PFDoA | 77.38 | 83.43 |
| 10:2FTS | 78.13 | 82.53 |
| 6:6PFPi | 81.49 | 75.61 |
| N-EtFOSA-M | 116.52 | 74.86 |
| PFDoS | 75.35 | 81.66 |
| PFTrDA | 82.44 | 85.84 |
| 6:2diPAP | 76.87 | 85.21 |
| PFTeDA | 86.82 | 78.13 |
| 6:8PFPi | 83.82 | 74.34 |
| 6:2/8:2diPAP | 61.87 | 68.49 |
| PFHxDA | 79.47 | 82.66 |
| 8:2diPAP | 82.12 | 75.03 |
| PFODA | 86.92 | 87.32 |
| diSAmPAP | 74.31 | 96.15 |

* The compounds (HFPO-DA and FHEA) were not measured in the low QC, since the chosen concentration was lower than the LOD using our methodology.

Quality control (QC) samples were analyzed in triplicate on three different days. ^#^ a full list of abbreviations can be found in supplementary material **Table S1**. n.d., not detected
